# Nicotinic acetylcholine receptors in attention circuitry: the role of layer VI neurons of prefrontal cortex

**DOI:** 10.1007/s00018-013-1481-3

**Published:** 2013-10-12

**Authors:** Eliane Proulx, Matthew Piva, Michael K. Tian, Craig D. C. Bailey, Evelyn K. Lambe

**Affiliations:** 1 Department of Physiology, University of Toronto, 1 King’s College Circle, Toronto, ON M5S 1A8 Canada; 2 Department of Cellular and Physiological Sciences, University of British Columbia, Vancouver, BC Canada; 3 Department of Biomedical Sciences, Ontario Veterinary College, University of Guelph, Guelph, ON Canada; 4 Department of Obstetrics and Gynaecology, University of Toronto, Toronto, ON Canada

**Keywords:** Nicotinic acetylcholine receptors, Attention, *chrna5*, Medial prefrontal cortex, Electrophysiology

## Abstract

Cholinergic modulation of prefrontal cortex is essential for attention. In essence, it focuses the mind on relevant, transient stimuli in support of goal-directed behavior. The excitation of prefrontal layer VI neurons through nicotinic acetylcholine receptors optimizes local and top-down control of attention. Layer VI of prefrontal cortex is the origin of a dense feedback projection to the thalamus and is one of only a handful of brain regions that express the α5 nicotinic receptor subunit, encoded by the gene *chrna5*. This accessory nicotinic receptor subunit alters the properties of high-affinity nicotinic receptors in layer VI pyramidal neurons in both development and adulthood. Studies investigating the consequences of genetic deletion of α5, as well as other disruptions to nicotinic receptors, find attention deficits together with altered cholinergic excitation of layer VI neurons and aberrant neuronal morphology. Nicotinic receptors in prefrontal layer VI neurons play an essential role in focusing attention under challenging circumstances. In this regard, they do not act in isolation, but rather in concert with cholinergic receptors in other parts of prefrontal circuitry. This review urges an intensification of focus on the cellular mechanisms and plasticity of prefrontal attention circuitry. Disruptions in attention are one of the greatest contributing factors to disease burden in psychiatric and neurological disorders, and enhancing attention may require different approaches in the normal and disordered prefrontal cortex.

Attention has been eloquently described as the ‘searchlight’ that focuses on relevant information in the midst of distraction in order to support goal-directed behavior [1]. In particular, it plays a pivotal role in mediating the executive functions of the prefrontal cortex [2, 3], a site of sensorimotor and emotional integration that is uniquely positioned to execute top-down control permissive to the orchestration of complex, flexible, and purposeful behavior such as problem solving, planning, and decision making [1, 3–5]. Given its intimate relationship to awareness, attention has also been qualified as the gateway to consciousness [2, 3, 6, 7].

Acetylcholine has long been known to play a role in cognition [8–10]. Non-specific lesions of the cholinergic neurons of the basal forebrain first suggested a more specific involvement of acetylcholine in attention [11–16], and it subsequently became clear that cholinergic projections to the prefrontal cortex are especially important in this regard [17, 18]. The importance of cholinergic modulation of prefrontal cortex can be seen in the detrimental effects for attention of specific lesions to its cholinergic projections. These projections, as shown in the schematic in Fig. 1,

**Fig. 1:**
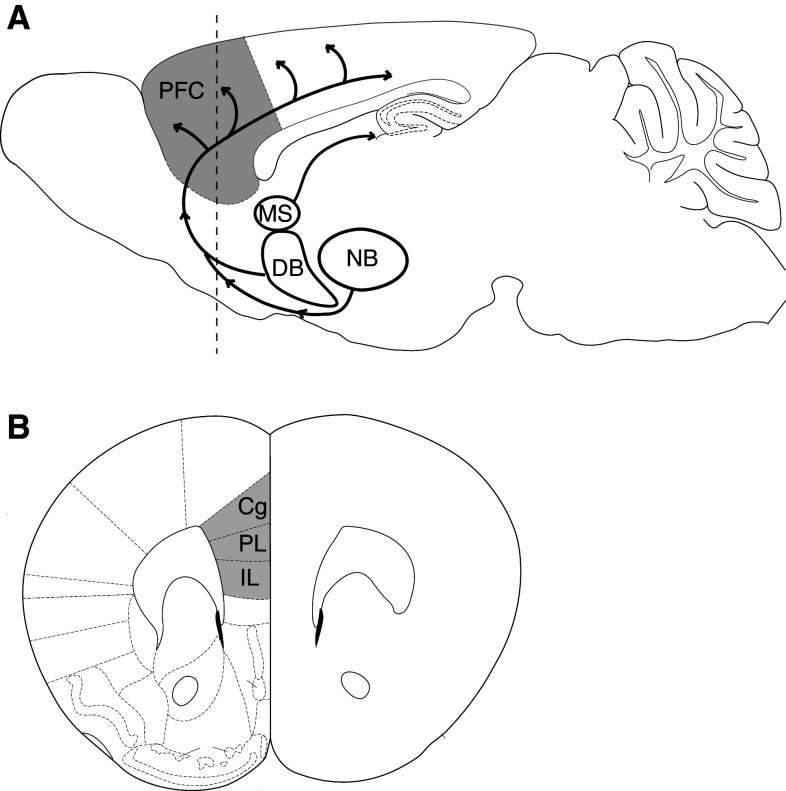
**a** The medial prefrontal cortex shown in gray receives cholinergic innervation from the basal forebrain. Figure adapted from Woolf [25] and Paxinos and Franklin [277] and is based on findings from Rye et al., Luiten et al., and Gaykema et al. [23, 160, 278]. The *dashed line* indicates the approximate location of the coronal section shown below. **b** Coronal brain section showing the subregions of rodent medial prefrontal cortex (in *gray*). *Cg* cingulate cortex, *DB* diagonal band, *IL* infralimbic cortex, *MS* medial septal nucleus, *NB* nucleus basalis, *PFC* prefrontal cortex, *PL* prelimbic cortex

include a dense cholinergic innervation from the basal forebrain, principally from the basal nucleus and parts of the diagonal band, but also from the magnocellular preoptic nucleus and substantia innominata [19–26]. Intrabasalis infusions of the cholinergic immunotoxin 192 IgG-saporin lead to the loss of cortical cholinergic afferents, reduced acetylcholine efflux in the prefrontal cortex, and significant impairments on attention tasks [27, 28]. Bilateral infusions of 192 IgG saporin in medial prefrontal cortex are equally detrimental and demonstrate that its deafferentation of cholinergic projections is sufficient to produce attentional impairments [17, 18, 29].

The importance of prefrontal cholinergic modulation was further suggested by microdialysis studies showing robust acetylcholine efflux within the prefrontal cortex during the performance of attention tasks [30–32], which reflects both attentional effort [33, 34] and behavioral context [35]. Moreover, the development of choline-sensitive microelectrodes, which offer greater temporal resolution than microdialysis probes, has further revealed that acetylcholine release in the prefrontal cortex increases rapidly and transiently—on the timescale of seconds to minutes—during the performance of attention tasks [29] where, as we will emphasize in this review, it can exert profound effects on corticothalamic neurons via the nicotinic acetylcholine receptors [36–38].

## Layer VI corticothalamic neurons of the prefrontal cortex play a central role in attention

Acetylcholine optimizes prefrontal cortical circuitry for top-down control [39–41]. Corticothalamic neurons, which constitute a large proportion of layer VI pyramidal cells [42], are uniquely positioned to exert these top-down influences and are robustly excited by acetylcholine [36]. These neurons integrate highly processed information from layer V pyramidal cells, from layer VI cortico-cortical neurons, and from direct thalamic inputs [42]. In turn, they exert powerful feedback influences on the thalamus [43–46]. While not all neurons in layer VI are corticothalamic, it is important to note that there are ten times more corticothalamic feedback projections than there are thalamocortical afferents [47], such that cholinergic modulation of these neurons will exert important influences on the circuits of attention.

Layer VI corticothalamic neurons constitute the major source of excitatory afferents to the thalamus [48], where they affect both the inhibitory reticular thalamic neurons [49] and the excitatory thalamocortical projection neurons [50]. During the tonic firing of wakefulness, the overall effect of this corticothalamic feedback is to *focus* thalamic and thalamocortical excitation [51], in part by modulating the sensitivity of thalamic neurons to incoming sensory stimuli [48, 52–54]. Prefronto-thalamic connectivity is further privileged in its modulation of attention due to its relationship with the midline and intralaminar thalamic nuclei that have long been implicated in awareness and attention [54–58].

The high percentage of layer VI neurons responding to acetylcholine [59] suggests that corticothalamic neurons are not the exclusive population of neurons subject to cholinergic modulation. This point should be emphasized since recent work has shown that layer VI neurons as a class exert powerful gain control over all the other cortical layers [60]. Cholinergic innervation is present in all layers of the prefrontal cortex [24, 26], but appears biased toward activation of the deepest layers [61]. Clear labeling of cholinergic fibers is observed in the deep cortical layers [24, 26], as demonstrated with immunostaining for choline-acetyltransferase (ChAT), the enzyme that catalyzes the synthesis of acetylcholine from acetyl-CoA and choline. Furthermore, anterograde labeling of ChAT positive cholinergic afferents from the basal forebrain indicate preferential projection to deep layers V/VI [62]. The apical dendrites from a large fraction of layer VI neurons extend all the way to the pial surface [63], where they may also be stimulated by cholinergic projections (and possibly also by cholinergic interneurons [64]) in superficial layers II/III [26, 64].

## Nicotinic acetylcholine receptors and their modulation of prefrontal layer VI neurons

The neurotransmitter acetylcholine acts on two classes of receptors—the ionotropic nicotinic receptors, which are the main focus of this review, and the metabotropic muscarinic acetylcholine receptors, which are G-protein coupled. Nicotinic acetylcholine receptors are pentameric ligand-gated cation channels [65, 66], permeable to Na^+^, K^+^, and Ca^2+^ ions [65, 67]. Two families of subunits can contribute to the pentameric structure necessary for functional nicotinic receptors: the α subunits (α2–α10) and the β subunits (β2–β4) [65, 66, 68]. They are arranged in a pinwheel around a central pore, assembled either as α-containing homomers or α/β heteromers. Nicotinic receptors are widely expressed in the central nervous system, and subunit composition differs from one region to the next [65, 66]. The subunit composition and stoichiometry of nicotinic receptors influence their functional properties, with important implications for nicotinic signaling [37, 69–72].

The most widely expressed nicotinic acetylcholine receptors in the brain are the α4β2-containing receptors (α4β2*) [65, 73–75], which are prominently expressed throughout cortex [76–79]. The homomeric α7 nicotinic receptors are also expressed in cortex, although only weak labeling has been documented in cortical layer VI [80]. Interestingly, while the α4, α5, α7, and β2 nicotinic receptor subunits show similar expression patterns in rodent and primate brain [81], there are some species differences in the expression of nicotinic receptors with potential implications for cholinergic modulation of attention circuitry. For example, the α2 nicotinic subunit is only widely expressed in primate brain [81], although it is not enriched in layer VI.

**Fig. 2:**
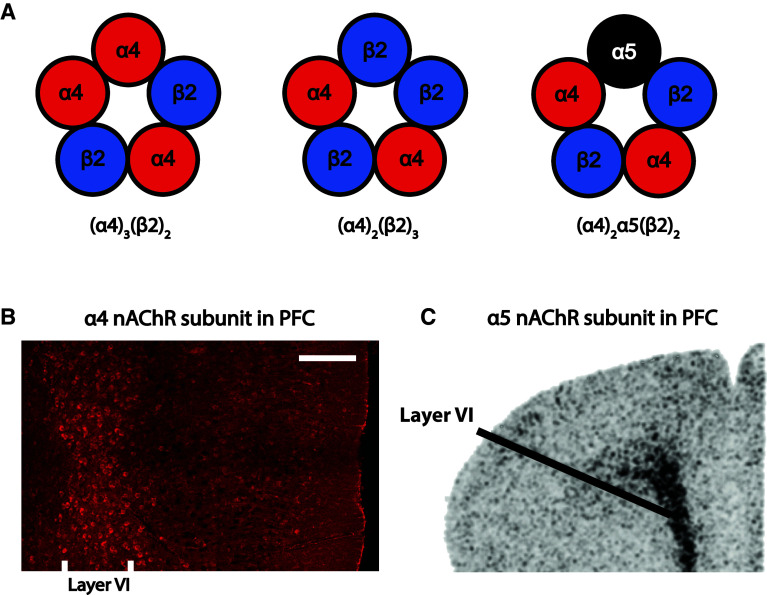
Subunit composition and layout of nicotinic acetylcholine receptor subunits in layer VI of medial prefrontal cortex. **a** Schematics showing three possible compositions of α4β2* nicotinic receptors within layer VI neurons of medial prefrontal cortex. Figure adapted from McKay et al. [279]. **b** Photomicrograph of mouse medial prefrontal cortex immunostained for YFP-tagged nicotinic acetylcholine receptor α4 subunits, putatively expressed in α4β2*-containing cells as shown at lower resolution by Marks and colleagues [93]. White matter on the right and the medial pial surface is on the left; adapted from Alves et al. [92]. *Scale bar* 200 μm. **c** In situ hybridization showing a dense band of α5 nicotinic subunit mRNA expression in layer VI of the medial prefrontal cortex; adapted from Wada et al. [86]

The α4β2* receptors have high affinity for nicotinic agonists (including acetylcholine and nicotine) and desensitize slowly, on the timescale of seconds [65, 82–84]. As illustrated in the schematic in Fig. 2,

the α4β2* nicotinic receptors can assume different stoichiometries, including (α4)_2_(β2)_3_ and (α4)_3_(β2)_2_. In the relatively rare brain regions that express the accessory α5 nicotinic subunit, such as layer VI of prefrontal cortex [86], these receptors can also incorporate the accessory α5 subunit to form (α4)_2_(β2)_2_(α5) receptors (α4α5β2) [65, 66, 85–88]. The accessory α5 subunits cannot form functional channels by themselves, since they do not contribute to the acetylcholine binding site and thus require co-assembly with other α and β subunits [65, 79]. However, inclusion of α5 can alter α4β2* nicotinic receptor properties substantially [71, 87, 88]: it can enhance receptor assembly and expression [87, 89], modulate receptor sensitivity to acetylcholine [37, 65, 69, 88, 90, 91], increase Ca^2+^ permeability [88], and confer sensitivity to allosteric modulation by galanthamine [36, 88].

Immunohistochemistry for YFP-tagged nicotinic α4 subunits in a knockin mouse suggests that high-affinity nicotinic receptors are densely expressed in layer VI of prefrontal cortex [92], where the accessory α5 subunit is also prominently expressed [86, 93–95]. Interestingly, while only one-fifth of all α4β2* nicotinic receptors in the brain are estimated to contain the α5 accessory subunit [65, 89, 96], prefrontal layer VI nicotinic receptors appear to incorporate α5 to a disproportionately large extent [37]. Indeed, functional concentration–response analyses of prefrontal corticothalamic neurons from WT and α5 knockout mice (α5^−/−^) suggest that the vast majority of α4β2* nicotinic receptors of its layer VI neurons are affected by this subunit [37]. As we will see, this unique expression pattern has ramifications for attentional signaling and behavior [37].

During the performance of attention tasks, brief transients of acetylcholine are released in medial prefrontal cortex [97, 98]. Population calcium imaging in slices of prefrontal cortex has demonstrated that nicotinic receptor stimulation by acetylcholine predominantly activates neurons within the deep cortical layers V/VI [61]. At the cellular level, acetylcholine elicits robust excitatory responses in the layer VI corticothalamic neurons of the medial prefrontal cortex that appear to be directly mediated by stimulation of somatodendritic postsynaptic α4α5β2 nicotinic receptors [36, 37, 59]. Acetylcholine binding to the nicotinic receptor leads to rapid conformational changes that result in channel opening and the flow of Na^+^, K^+^, and Ca^2+^ cations through the pore [65, 66, 83]. Nicotinic receptors rectify at more depolarized membrane potential [99, 100], such that acetylcholine likely exerts more profound effects near the resting membrane potential, where the effect of nicotinic stimulation is excitatory and results in depolarization. When sufficiently large, this membrane depolarization can lead to the generation of action potentials. Acetylcholine depolarizes the vast majority of layer VI pyramidal cells in this way [36], but these excitatory nicotinic responses are completely eliminated in β2^−/−^ mice [38, 59], which lack functional α4β2* nicotinic receptors, and are significantly reduced in α5^−/−^ mice [59].

**Fig. 3:**
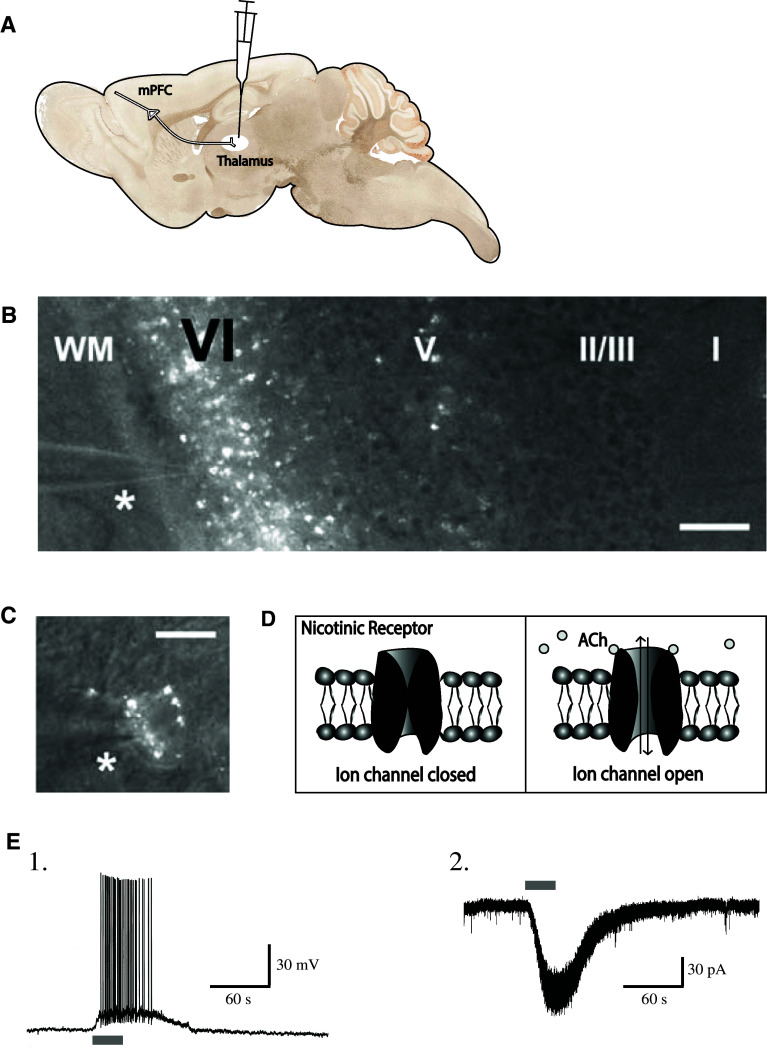
Acetylcholine (ACh) excites labeled corticothalamic neurons in layer VI of medial prefrontal cortex. **a** Retrograde labeling of corticothalamic neurons through in vivo stereotaxic surgery to inject rhodamine microspheres into the medial dorsal thalamus. **b** Prominent retrograde labeling of layer VI neurons in a coronal prefrontal brain slice. The *asterisk* marks the location of a patch pipette for electrophysiologcal recordings. *Scale bar* 240 μm. Figure adapted from Kassam et al. [36]. **c** A high-magnification view of a labeled pyramidal cell body. *Scale bar* 20 μm. Figure adapted from Kassam et al. [36]. **d** Schematic showing the closed and open states of the nicotinic acetylcholine receptor. **e** A retrograde-labeled corticothalamic neuron in layer VI of medial prefrontal cortex responds to acetylcholine in (*1*) current clamp and (*2*) voltage clamp. Figure adapted from Kassam et al. [36]

The nicotinic responses that result from the current carried by the flow of Na^+^, K^+^, and Ca^2+^ ions through the nicotinic receptor pore can be examined electrophysiologically in voltage clamp (where the membrane potential can be held constant experimentally so as to allow the measurement of the nicotinic current) or in current clamp (where the membrane potential is allowed to fluctuate and the injected current, or lack thereof, is held constant). Figure 3 illustrates

the robust excitatory effects of nicotinic stimulation that were recorded in retrogradely labeled corticothalamic neurons in slices of prefrontal cortex [36]. Since corticothalamic neurons can also be distinguished from cortico-cortical cells based on electrophysiological properties [101, 102], Kassam et al. [36] were further able to establish that nicotinic stimulation exerts more profound excitation of cortico-thalamic than cortico-cortical neurons of layer VI prefrontal cortex.

The excitatory nicotinic responses of layer VI pyramidal neurons are directly mediated by postsynaptic somatodendritic receptors since currents are resistant to blockade of synaptic transmission by the Na^+^ channel antagonist tetrodotoxin and to pharmacological inhibition of ionotropic and metabotropic glutamate receptors [36]. Pharmacologically, these nicotinic currents are suppressed by the α4β2* competitive antagonist DHβE, insensitive to the α7 antagonist MLA and potentiated by the α5 allosteric modulator galanthamine [36, 88]. These findings are consistent with α4α5β2 nicotinic receptor involvement [36]. Most convincing, however, is the demonstration that nicotinic excitation of layer VI pyramidal cells is substantially reduced in mice in which the α5 subunit has been genetically deleted (α5^−/−^) [37]. Together, these findings highlight that the relatively rare α5 subunit plays an important role in mediating optimal cholinergic excitation of layer VI neurons of the prefrontal cortex, where it is densely expressed and incorporated into α4β2* nicotinic receptors.

## Nicotinic receptors and attentional performance

**Fig. 4:**
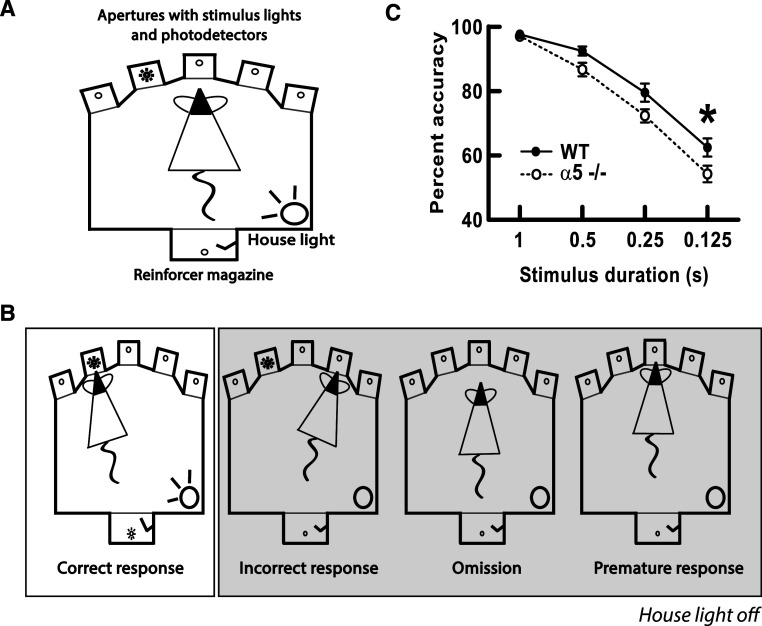
Under challenging conditions, mice lacking the nicotinic α5 subunit (α5^−/−^) respond with decreased accuracy relative to wild-type (WT) mice in the 5-choice serial reaction time task (5-CSRTT). **a** Schematic of the operant chamber for the 5-CSRTT. **b** Four typical responses of mice performing the 5-CSRTT. From *left* *to* *right*: the correct response, the incorrect response, an omission, and a premature response. Figure adapted from Dalley et al. [280]. **c** Nicotinic receptor α5^−/−^ mice perform significantly worse than wild-type controls in the 5-CSRTT when stimulus duration is brief. Figure adapted from Bailey et al. [37]

At the behavioral level, the α5 subunit is required for normal attention performance under challenging conditions [37]. The five-choice serial reaction time task (5-CSRTT) is a commonly used attention task that involves sustained and divided attention [103]. Briefly, the animal is placed in an operant chamber, illustrated in Fig. 4.

A light stimulus, whose duration can be varied to alter the difficulty of the attention task, is randomly flashed in one of five apertures. The animal is required to attend to, and subsequently accurately recall, the location of this stimulus within a fixed time period. Attention performance is assessed by correct identification of the location of the stimulus by nose poke. This task measures various aspects of attentional control, including accuracy (correct responses), omissions (lack of response, reflects inattentiveness), perseveration (repeated responses at the same location, reflects lack of flexibility), and premature responses (responding before the end of the inter-trial interval, reflects impulsivity). The α5^−/−^ mice show deficits in accuracy on the 5-CSRTT when stimulus duration is brief, a condition that requires greater attentional demand, but perform normally under baseline training conditions, when stimulus duration is longer. Interestingly, equivalent deficits in attention performance in humans are highly disruptive to cognitive function [104–107]. Mice lacking the β2 subunit (β2^−/−^) also show significant impairments on the 5-CSRTT, and these deficits can be rescued by lentiviral vector-mediated re-expression of β2-containing nicotinic receptors in the prefrontal cortex [38]. The 5-CSRTT studies in α5^−/−^ and β2^−/−^ mice employed different training and testing approaches, which may explain subtle differences in the nature of the attention deficit observed [37, 38].

## Compensatory plasticity of cholinergic responses in prefrontal layer VI neurons

The question arises whether the differences in attention performance observed in α5^−/−^ mice result completely from the impaired nicotinic stimulation of α4α5β2-containing nicotinic receptors within corticothalamic circuits of adult prefrontal cortex or whether the loss of this nicotinic stimulation leads to functional or structural alterations of attention circuitry. It is conceivable that plasticity in the cholinergic system might ameliorate attention deficits that might otherwise be more severe; for example, allowing α5^−/−^ mice to perform at near-normal levels of accuracy when longer stimulus durations are used in the 5-CSRTT [37].

**Fig. 5:**
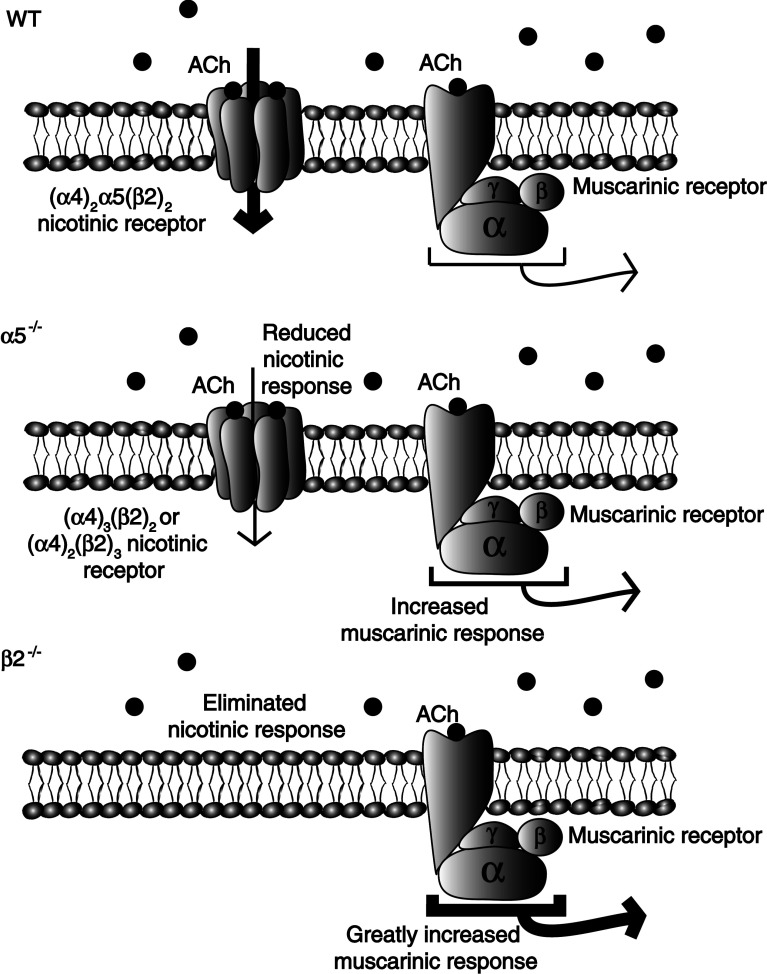
Plasticity between nicotinic and muscarinic acetylcholine (ACh) receptors in layer VI neurons of medial prefrontal cortex. Typical responses in layer VI pyramidal neurons are highly driven by nicotinic receptors, whereas muscarinic effects are less prominent. In knockout mice with decreased nicotinic receptor function, muscarinic responses are enhanced. This compensatory upregulation in muscarinic receptor function is apparent in α5^−/−^ mice and very pronounced in β2^−/−^ mice. Figure summarizing results from Tian et al. [59]

We have observed that cholinergic excitation of the layer VI pyramidal cells primarily involves nicotinic receptors in wild-type mice [59]; however, genetic deletion of the nicotinic α5 or β2 subunits (α5^−/−^ and β2^−/−^, respectively) leads to the compensatory upregulation of muscarinic acetylcholine receptor excitation [59]. These G-protein coupled receptors couple to second messenger cascades and exert slower excitatory actions, significantly changing the mechanisms and timing of the cholinergic response in these layer VI neurons [59]. A schematic of this compensatory plasticity is shown in Fig. 5;

it appears to affect neurons from β2^−/−^ mice to a greater degree than those from α5^−/−^ mice [59]. This unusual plasticity of layer VI cholinergic responsiveness indicates that the attention impairments associated with disruption of nicotinic signaling are more complex than originally anticipated. It is unclear at what stage of maturation this plasticity occurs and whether it can be reversed given sufficient time after adult rescue of the missing nicotinic receptor subunits [38].

## Nicotinic receptor α5 subunit and morphological maturation of prefrontal layer VI neurons

The maturation of executive function and attention requires the normal development of prefrontal cortex [108–110], and developmental lesions of the cholinergic system disrupt neuronal morphology and cortical circuitry [111–114]. Cortical nicotinic acetylcholine receptors play an important role in the development of attention circuitry [36, 63, 115], and aberrations in cortical nicotinic binding are reported to occur in many neurodevelopmental disorders, including autism [116, 117], epilepsy [118], and schizophrenia [119, 120].

Cholinergic innervation of the prefrontal cortex is well developed by the third week of postnatal life in rodents [121, 122], a time period equivalent to the perinatal period in humans [123, 124]. Dense ChAT immunostaining can be seen in the frontal cortex at this time [122], and high levels of α4β2* nicotinic binding are observed in prefrontal layer VI [125]. Furthermore, peak mRNA levels for the α5 subunit are seen in layer VI during the first 2–3 weeks of postnatal development [95]. By contrast, cortical mRNA levels for the α4 and β2 subunits show a somewhat different pattern with a peak at birth and a slight decline before maintaining relatively constant expression across postnatal development [126, 127].

**Fig. 6:**
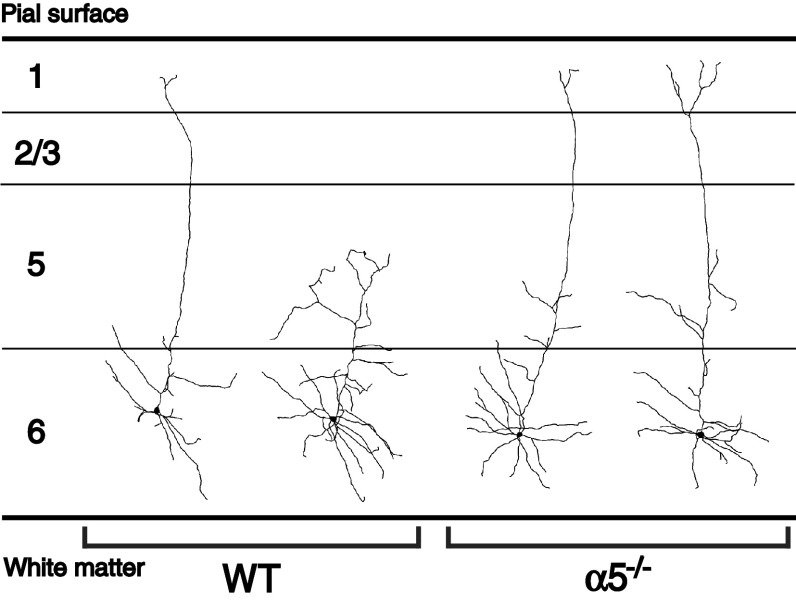
The morphology of layer VI neurons in medial prefrontal cortex differs between wild-type and α5^−/−^ mice. In adult wild-type mice, there is a roughly equal distribution of layer VI pyramidal neurons that have long apical dendrites that terminate at the pial surface and those that have short apical dendrites that terminate within the mid-layers of the medial prefrontal cortex. In contrast, layer VI neurons of α5^−/−^ mice show a preponderance of neurons with long apical dendrites. In this sense, it could be said the layer VI neurons of α5^−/−^ mice retain a developmental phenotype in the pattern of their apical dendritic morphology. In young mice of both genotypes, layer VI neurons have only long apical dendrites. Figure adapted from Bailey et al. [63]. Of note, these morphological changes can be recapitulated in wild-type mice by chronic in vivo nicotine treatment during development [281], likely mediated through desensitization of nicotinic receptors [281]

Developmental differences in nicotinic excitation and dendritic morphology coincide temporally with changes in α5 expression. The excitatory nicotinic currents of layer VI neurons exhibit a developmental profile, peaking within the first postnatal month [36]. Nicotinic stimulation can influence neuronal morphology and spur neurite retraction [128, 129], and in the first morphological analysis of these cells, Bailey et al. [63] showed that key developmental changes in neuronal complexity appear to be initiated within this critical time period. Specifically, there appears to be a developmental retraction of the apical dendrites of layer VI prefrontal cortex: whereas almost all the apical dendrites of layer VI pyramidal neurons extend to the pial surface in young mice at postnatal week 3, half of them terminate in the mid-layers by adulthood [63]. As illustrated in Fig. 6,

these maturational changes in the dendritic morphology of layer VI neurons are absent in the α5^−/−^ mice, without any further differences in overall cortical morphology [63]. Furthermore, layer VI neurons of α5^−/−^ mice show negligible developmental changes in nicotinic excitation [63]. Thus, the α5 subunit appears to be essential for the normal maturation of corticothalamic circuitry and drives developmental differences in layer VI excitation and morphology.

In summary, there are extensive differences between WT and α5^−/−^ mice in development and adulthood. These differences are relevant to the deficits in attention performance seen in α5^−/−^ mice in adulthood and are summarized in Table 1.

**Table 1:** Categories of differences between WT and α5^−/−^ mice

Effects	WT	α5^−/−^
Neuropharmacology in layer VI pyramidal cells [37, 59, 63]		
ACh-elicited nicotinic receptor currents (1 mM)	40 ± 5 pA	14 ± 1 pA*
Nicotine-elicited nicotinic receptor currents (300 nM)	16 ± 2 pA	6 ± 1 pA*
Desensitization (% decrease) of ACh response after nicotine	36 ± 4 %	73 ± 4 %*
ACh-elicited muscarinic depolarization from rest	2.9 ± 0.5 mV	6.5 ± 1.3 mV*
ACh-elicited muscarinic increase in spiking frequency in excited state	309 ± 23 %	462 ± 65 %*
Developmental changes in ACh-induced currents	Peak in young mice	No change*
Dendritic morphology of layer VI pyramidal cells [63]		
Young mice: % apical dendrites extending to the pial surface	82 %	92 %
Adult mice: % apical dendrites extending to the pial surface	45 %	92 %*
Attention behavior [37]		
Performance accuracy on non-demanding attention tasks	98 ± 1 %	97 ± 1 %
Performance accuracy on demanding attention tasks	63 ± 3 %	54 ± 3 %*
Systemic nicotine changes attentional accuracy on demanding tasks	−5 ± 1 %**	−1 ± 4 %

Data are shown as mean ± SEM (where appropriate)

* Indicates a statistically significant difference from WT with *P* < 0.05

** Indicates a statistically significant change from baseline with *P* < 0.05

## Sex differences in nicotinic excitation of layer VI neurons during postnatal development

Interestingly, there are also developmental sex differences in nicotinic excitation [92]. Prefrontal layer VI nicotinic currents show a similar developmental profile in males and females, with peak nicotinic excitation achieved around the 3rd week of postnatal life and declining by the 5th week. However, within the 1st postnatal month, nicotinic currents are larger and observed in a greater proportion of cells in males than in females. It is not known whether there are any sex differences in α5 expression or function, although it appears that a similar percentage of layer VI neurons express α4 nAChRs in developing male and female mice [92]. In fact, this sex difference in nicotinic excitation of layer VI neurons during postnatal development may arise from differences in cortical neurosteroid levels between males and females. The sex steroid progesterone, for example, can directly suppress nicotinic currents through negative allosteric modulation of α4β2* nAChRs [130, 131]. The pre-pubertal rodent brain expresses all the enzymes necessary for the de novo synthesis of progesterone from cholesterol [132, 133], and the rate-limiting enzyme in this pathway shows a trend toward greater cortical expression in females than males at this stage of development [132]. Furthermore, evidence suggests that estrogenic steroid hormones may directly interact with the nicotinic receptor to potentiate excitatory ACh responses [134]. Developmental sex differences in the maturation of attention circuitry may help account for vulnerability to attention deficit disorders, which are twice as prevalent in males than females [135–137].

## Additional mechanisms of cholinergic modulation of prefrontal cortex

Although nicotinic receptors located on pyramidal neurons in layer VI of the medial prefrontal cortex play a critical role in mediating attentional processes, they do not act in isolation. There are cholinergic receptors on other prefrontal neurons and on neurons in other brain regions that also contribute to attentional processing in prefrontal cortex. Relevant cholinergic receptors within prefrontal cortex itself include those on layer V neurons, on the terminals of thalamocortical projections, monoaminergic projections, and on cortical interneurons.

Acetylcholine exerts layer-specific effects in the prefrontal cortex [61], and although nicotinic stimulation exerts many effects across the prefrontal cortical column, it appears to enhance preferentially deep layer activation [61]. An elegant optogenetic study by Olsen et al. [60] has recently demonstrated that in the visual cortex, activation of layer VI cells exerts powerful gain control by means of feedback inhibition of the cortical column. It is tempting to speculate that preferential activation of the deep layers of prefrontal cortex by acetylcholine facilitates such information processing. As we have seen, layer VI pyramidal neurons show a robust excitatory response to acetylcholine mediated by postsynaptic somatodendritic nicotinic receptors [36, 37]. In contrast to layer VI, the layer V pyramidal neurons of the prefrontal cortex are predominantly subject to muscarinic modulation [138], although a rapid α7-mediated nicotinic response has been documented in the prefrontal cortex of juvenile mice [61]. Importantly in this layer, α4β2*-containing nicotinic receptors on thalamocortical terminals strongly facilitate thalamic excitation of layer V pyramidal neurons [139–141], an indirect effect that translates into a large increase in the frequency of rapid, glutamatergic excitatory postsynaptic currents. Of note, a positive feedback relationship has been demonstrated between nicotinic-elicited prefrontal glutamatergic release and the release of acetylcholine itself from cholinergic terminals in prefrontal cortex [97, 98, 142]. Nicotinic receptors have also been implicated in the modulation of monoamine release in the prefrontal cortex [143–145].

Nicotinic modulation of prefrontal GABAergic interneurons also likely contributes to attentional processing. Although α4β2*- and α7-containing nicotinic receptors excite only limited subpopulations of interneurons in the cerebral cortex [146, 147], many layer-specific effects have been documented. In layer VI, fast-spiking interneurons are excited indirectly by nicotinic stimulation [36], presumably due to innervation by corticothalamic axon collaterals [101]. In layer V, stimulation of nicotinic receptors on GABAergic interneurons increases the frequency of inhibitory postsynaptic currents on pyramidal neurons [148, 149], promotes intracolumnar inhibition [150], and modulates spike timing-dependent synaptic plasticity [149]. Most pyramidal neurons in layer II/III do not contain nicotinic receptors, nor do they receive glutamatergic inputs subject to nicotinic modulation ([61], but see [151, 152]). Instead, nicotinic receptors are found on interneurons that exert feedforward inhibition onto layer II/III pyramidal cells [61]. Nicotinic stimulation of the superficial layer I interneurons enhances synchronous activity of inhibitory cortical networks in superficial cortex [153, 154].

## Nicotinic receptor and prefrontal attention circuitry in health and disease

The prefrontal cortex is a critical node in widespread and dynamic brain networks that sustain higher cognitive function in health and that perpetuate executive dysfunction in psychiatric illness [155, 156]. The cholinergic modulation of prefrontal cortex is especially powerful in its ability to subsequently influence downstream cortical and subcortical networks [4, 157, 158], as well as being uniquely positioned to exert feedback control on neuromodulatory centers [159], including the cholinergic nuclei [160, 161]. Neuroimaging studies have revealed that the prefrontal cortex is consistently activated on attention tasks, often in conjunction with the parietal cortex [162–165], which is recruited by the prefrontal cortex under conditions of increased attentional demand [157].

A substantial body of work addresses the effects of acetylcholine on attention by manipulating endogenous levels of acetylcholine and by pharmacologically or genetically altering nicotinic acetylcholine receptors. Indeed, many genetic and pharmacological studies using both animal models and human subjects have found that nicotinic acetylcholine receptors are of particular importance for attention, as summarized in Table 2.

**Table 2:** Nicotinic receptor effects on attention

Manipulation	Species	Task	Effects on attention	References
**Genetic studies**				
α5 subunit KO	Mice	5-CSRTT	↓	[37]
β2 subunit KO	Mice	5-CSRTT	↓	[38]
α7 subunit KO	Mice	5-CSRTT	↓	[166, 167, 234]
	Mice	5-CSRTT	–	[38]
**Human polymorphisms** (arrow indicates effect of the risk allele)				
α5 subunit	Humans	Selective and sustained attention (CPT)	↓	[168]
		n-back/CPT	↓	[235]
α4 subunit	Humans	ADHD inattentive symptoms	↓	[236]
		Cued visual search task	↓	[237]
		Selective and sustained attention (CPT)	↓	[168]
		Multiple object tracking and visual search	↓	[238]
β2 subunit	Humans	Selective attention (CPT)	↓	[168]
α7 subunit	Humans	Sustained attention (CPT)	↑ in smokers ↓ in nonsmokers	[168]
**Lesion studies**				
Basal forebrain lesions	Rats	5-CSRTT	↓	[13, 176, 239, 240]
Nucleus basalis of Meynert lesions	Rats	5-CSRTT	↓	[28, 31, 32]
mPFC lesions	Rats	5-CSRTT	↓	[241, 242]
mPFC lesions	Rats	Attentional set-shifting	**↓**	[243]
Lesions of PFC cholinergic fibers	Rats	5-CSRTT	**↓**	[17]
Lesions of PFC cholinergic fibers	Rats	SAT/dSAT	**↓**	[18]
**Pharmacological studies**				
* Nicotine* (agonist of nicotinic receptors, but act as an antagonist by desensitization)				
Nicotine	Monkeys	Covert orienting	↑	[244]
Nicotine	Monkeys	DMTS-D	↑	[175]
Nicotine	Rats	5-CSRTT	↑	[245]
Nicotine	Rats	5-CSRTT	–	[246]
Nicotine	Rats	Stimulus detection	↑	[178, 247–249]
Nicotine	Rats	5-CSRTT	↑	[180, 182, 250–253]
Nicotine	Rats (two strains)	5-CSRTT	↑ in Sprague–Dawley –in Lister	[177]
Nicotine	Rats	5-CSRTT	–(acute), ↑ (chronic)	[182]
Nicotine (local to HIP or mPFC)	Rats	5-CSRTT	–(HIP), ↑ (mPFC)	[180]
Nicotine	Mice	5-CSRTT	↑	[234]
Nicotine (local to mPFC)	Rats	3-CSRTT	↑ (mPFC)	[141]
Nicotine	Rats	5-CSRTT	↑ (acute and chronic)	[254]
Nicotine	Mice (three strains)	5-CSRTT	–(acute) ↑ (chronic) in all strains	[183]
Nicotine	Mice	5-CSRTT	↓	[37]
Nicotine	Rats	SAT	↓	[98]
Nicotine	Rats	Attention set-shifting	↑ (acute and sub-chronic)	[255]
Nicotine	Mice	5-CSRTT	↑	[256]
Nicotine (tablets)	Humans	Rapid info processing	↑	[186]
Nicotine (gum)	Humans	Two-letter/digit recall	↓	[191, 193]
Nicotine (subcutaneous)	Humans	Reaction time	–	[189]
Nicotine (subcutaneous)	Humans	Digit recall	↓	[192]
Nicotine (patch)	Humans	POMS/CPT/Digit recall	↑	[188]
Nicotine (gum)	Humans	Flight simulator	↑	[187]
Nicotine (patch)	Humans	Digit recall	–	[257]
Nicotine (patch)	Humans	Covert orienting	–	[258]
Nicotine (subcutaneous)	Humans	N-back	↑	[162]
Nicotine (gum)	Humans	ANT	–	[190]
Nicotine (gum)	Humans	Cue target detection	↑	[259]
Nicotine (gum)	Humans	Discrimination (Posner-type)	–	[260]
Nicotine (patch)	Humans	Stroop	–	[261]
Nicotine (gum)	Humans	Discrimination (Posner-type)	↑	[262, 263]
Nicotine (patch)	Humans	Multiple tasks	↑	[264]
Nicotine (gum)	Humans	RVIP	↑	[265]
Nicotine (patch)	Humans	Stroop/ANT	↑ (Stroop), ↓ (ANT)	[266]
Nicotine (intranasal)	Humans	CPT	↑	[267]
* Agonists of nicotinic receptors *				
ABT-418/ABT-089	Rats	DMTS-D	↑	[175, 176]
SIB-1533A	Rats	5-CSRTT	–	[250]
Dizocilpine then SIB-1533A	Rats	5-CSRTT	↓ (diz), attenuation with SIB	[268]
SIB-1533A	Monkeys	DMTS-D	↑	[268]
Epibatidine/ABT-418/isoarecolone/AR-R 17779	Rats	5-CSRTT	↑ (epi, ABT, iso), –(AR-R)	[180]
ABT-594/ABT-582941	Monkeys	DMTS-D	↑ (ABT-594, ABT-582941)	[269]
R3487/galanthamine	Rats	Signal detection	↑ (R3487), –(gal)	[270]
S 38232	Rats	SAT/dSAT	↑	[98]
ABT-594	Rats	5-CSRTT	↑	[271]
Dizocilpine/scopolamine then sazetidine-A	Rats	Signal detection	↓ (diz, sco), attenuation with saz	[179]
ABT-418	Mouse	5-CSRTT	↑	[256]
PNU 282987	Mouse	5-CSRTT	–	[256]
*Antagonists of nicotinic receptors*				
Mecamylamine	Rats	5-CSRTT	↓	[272]
Mecamylamine/hexamethonium	Rats	5-CSRTT	↓ (mec), –(hex)	[273]
Mecamylamine	Rats	Signal detection	↓	[178, 248]
Mecamylamine	Mice	5-CSRTT	↓ (mec) in three strains	[183]
Mecamylamine	Humans	Digit vigilance, RVIP	–(mec)	[274]
*Acetylcholinesterase inhibitors*				
Physostigmine	Rats	5-CSRTT	–	[272]
Donepezil	Humans	Flight simulator	↑	[275]
Donepezil	Humans	Anti-cueing	↑ (voluntary attention only)	[276]
*Acetylcholine reuptake blockers*				
Hemicholinium	Rats	5-CSRTT	↓	[13]

Knockout mouse strains for the α5, β2, and α7 nicotinic receptor subunits have all been found to display impaired attention performance on the 5-CSRTT [37, 38, 166, 167], and human subjects expressing genetic variations in the α5, α4, or β2 genes are associated with increased risk for nicotine dependence [168–174], which may in part develop as a result of attention deficits that promote early experimentation with drugs and alcohol [168, 170, 172]. Pharmacologically, various nicotinic agonists have been found to improve attention performance in animal studies [175–180], whereas nicotinic antagonists appear to disrupt attention [178, 181]. However, it is important to note that the effects of nicotine may depend on the history of nicotine exposure [182] and on strain/species differences [37, 183].

The agonist nicotine is an interesting example since it is selective for nicotinic receptors and has been used in a large number of animal and human studies. Overall, the effects of nicotine in humans are far more complex and controversial, with inconsistent effects on attention performance [184, 185]. While nicotine has also been shown to improve attention in humans [186–188], this is not always the case [189–193]. Evidence suggests that nicotine may have differential effects in human smoker and non-smoker populations [185, 194–196], and in patients with attention deficits [197, 198].

**Fig. 7:**
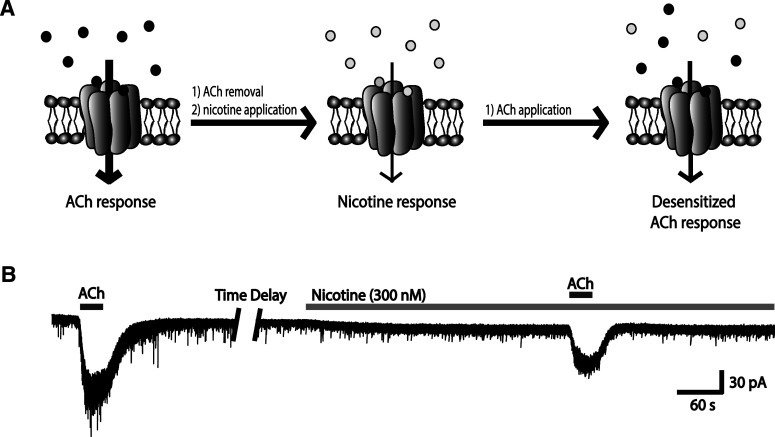
A concentration of nicotine similar to that seen in the blood of smokers markedly reduces subsequent nicotinic receptor-mediated responses to acetylcholine (ACh). **a** Schematic of the acetylcholine response, nicotine response, and acetylcholine response following receptor desensitization by nicotine. **b** Representative whole-cell recordings of a layer VI pyramidal neurons showing: *(1)* an initial response to ACh, *(2)* response to nicotine, and *(3)* response to ACh following desensitization by nicotine. Figure adapted from Bailey et al. [37]

At the cellular level, nicotinic receptors are subject to desensitization; that is, they can become temporarily inactive in the continued presence of agonist, leading to a reduction in response [83, 84]. Nicotine, at levels normally seen in the blood of smokers (~300 nM) [199–201], can have such an effect on α4β2* receptors [36, 37], as illustrated in Fig. 7.

Interestingly, Bailey et al. [37] reported that the α5 subunit normally protects against nicotine-induced desensitization, since layer VI neurons from WT mice show half as much desensitization as those of α5^−/−^ mice. The low-affinity α7* nicotinic acetylcholine receptors, on the other hand, do not appear to desensitize at these concentrations [202].

Deficits in attention have been reported in normal human aging [203] as well as a multitude of neurological and psychiatric disorders, such as Alzheimer’s disease and schizophrenia [204–207]. Decreases in prefrontal nicotinic receptor binding are observed in patients suffering from mild cognitive impairment [208, 209] as well as Alzheimer’s disease [210–215], and schizophrenia has been associated both with α7 subunit polymorphisms and expression changes [216, 217], as well as a with a higher incidence of the noncoding α5 nicotinic subunit polymorphism [218, 219]. What is more, nicotinic agonists of the α4β2* and α7 nicotinic receptors have been proposed as potential therapeutics for schizophrenia [220], Alzheimer’s disease [221–224], and attention deficit hyperactivity disorder [225–229].

## In conclusion

Layer VI nicotinic receptors are integral components of prefrontal attention circuitry in development and adulthood. Despite recent advances, there remains much to be understood about their effects on the maturation of the prefrontal cortex and the modulation of its neurons and networks. Fundamental questions about the regulation of nicotinic receptors in neurons of the living brain remain unanswered. An apparently large reserve of nicotinic receptors within layer VI prefrontal neurons [63, 92], for example, suggests the potential for targeted upregulation to the membrane [230, 231]. It is interesting to note that nicotinic receptor trafficking abnormalities have been documented in psychiatric illness [232]. The issue of physiological and structural plasticity [59, 63] further suggests that the brain may be fundamentally different in certain conditions, and the best treatments may not be those that would improve the performance of the normal brain. In this regard, it is essential for research to examine the realities of prefrontal attention circuitry in different conditions associated with attention deficits. These issues are all the more important to resolve given that nicotinic receptors in layer VI of prefrontal cortex are positioned to be potential drug targets in the treatment of the attention deficits associated with psychiatric and neurological diseases [233].
